# Bibliographical

**Published:** 1879-08

**Authors:** 


### BIBLIOGRAPHICAL.

### MECHANICAL DENTISTRY. BY CHARLES HUNTER.

*A practical treatise on the construction of the various kinds of artificial dentures, comprising also useful formulæ, tables and receipts for gold plate clasps, solders, etc., etc.*

This work made its appearance during the last year; it is an excellent presentation, in brief, of the principles of mechanical dentistry, just such an one as meets the requirements of the beginner. The description and directions are good, plain and to the point. It is also an excellent ready reference book for the practitioner.

The formulæ, tables and receipts in the latter part of the work are very valuable, and are quite accurate and reliable.

This book fills a long recognized want. It does not supercede, nor take the place of Dr. Richardson's Mechanical Dentistry, which discusses principles more fully.

The work is published by Lindsay & Blakiston, and as to its execution nothing further need be said. It is for sale by Robt. Clarke & Co., of this city; price $2.25. This work should be in the hands of every practitioner and student; for the latter it is a good text book.

